# Supplementary figures and images for: Comparative mitogenomics of Cheiracanthium species (Araneae: Cheiracanthiidae) with phylogenetic implication and evolutionary insights
Source: PeerJ. 2025 Feb 14;13:e18314. doi: 10.7717/peerj.18314 (PMC11831973; doi:10.7717/peerj.18314)

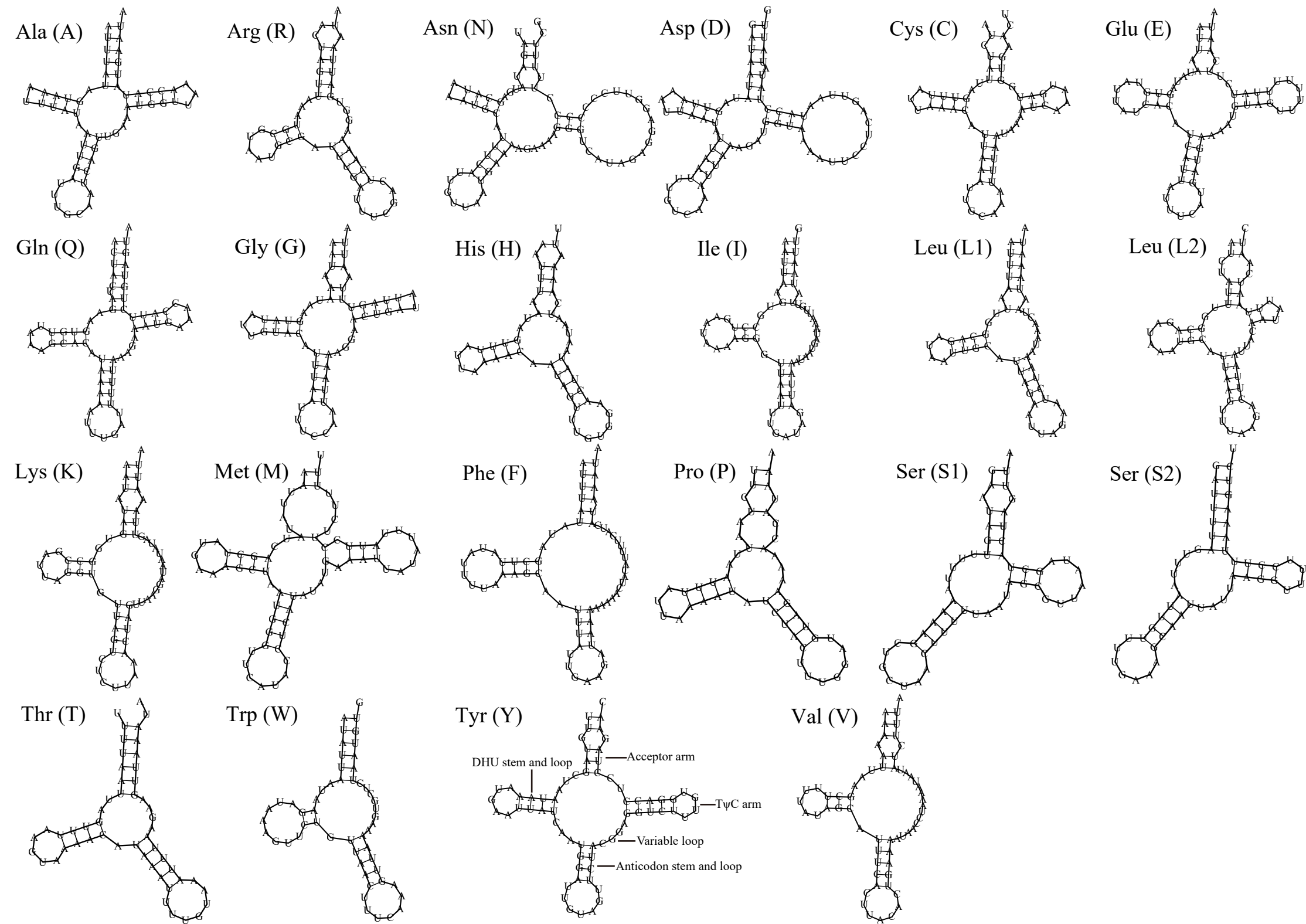

Supplement: Supplemental Information 5 — The tRNAs are labeled with the abbreviations of their corresponding amino acids. Names of structural components of a tRNA gene are indicated in the trnY structure. [file peerj-13-18314-s005.pdf]

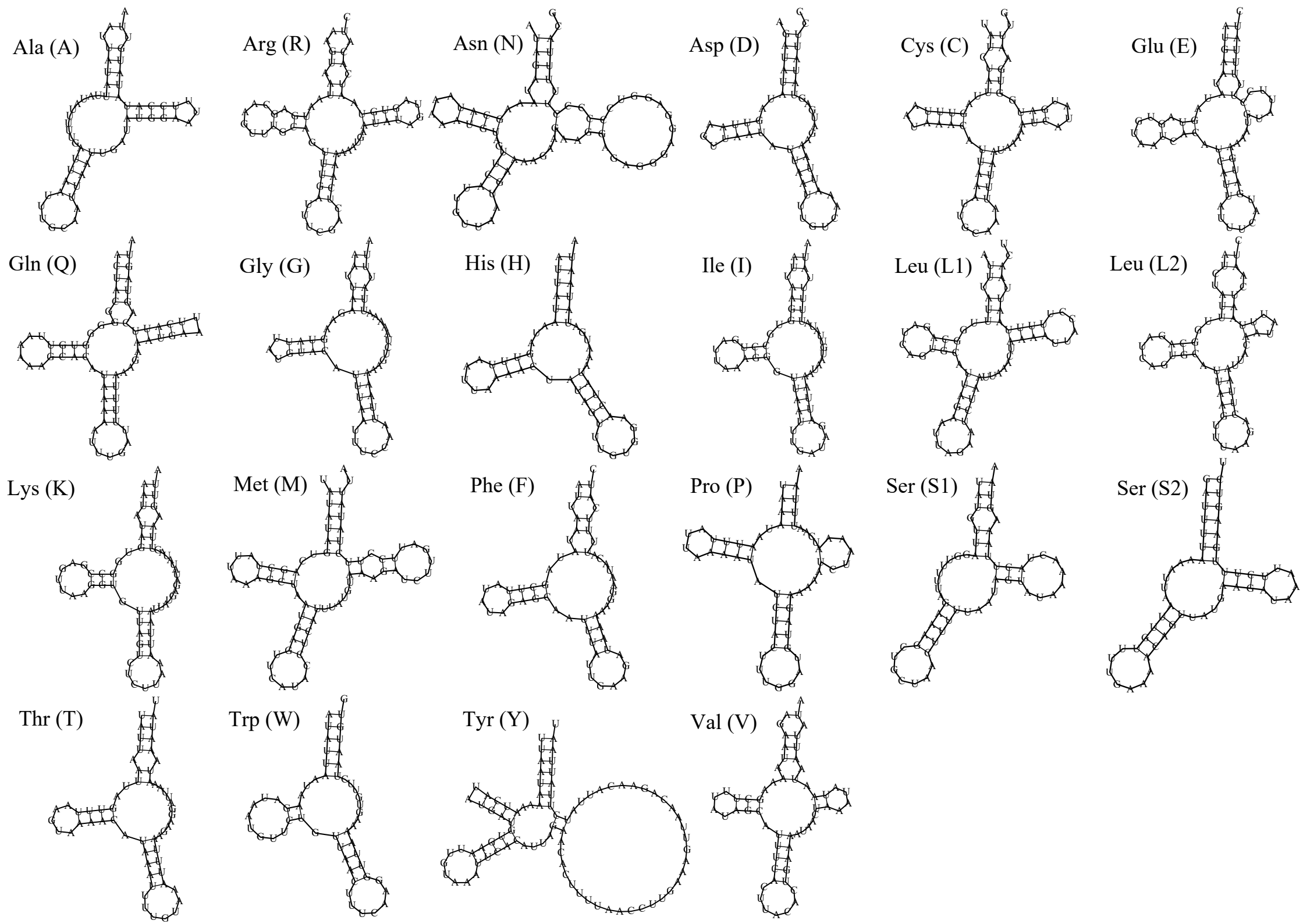

Supplement: Supplemental Information 6 — The tRNAs are labeled with the abbreviations of their corresponding amino acids. [file peerj-13-18314-s006.pdf]

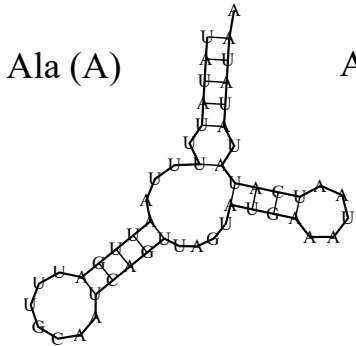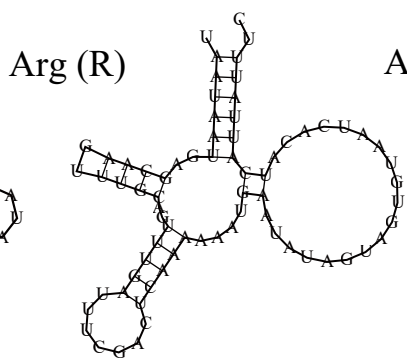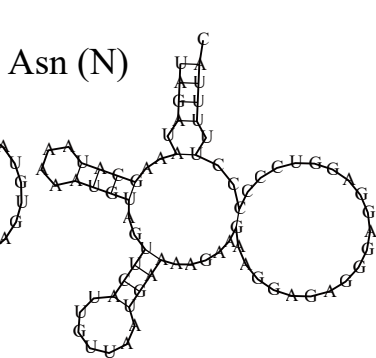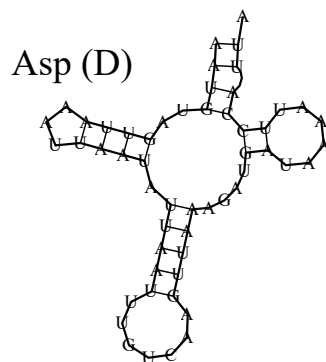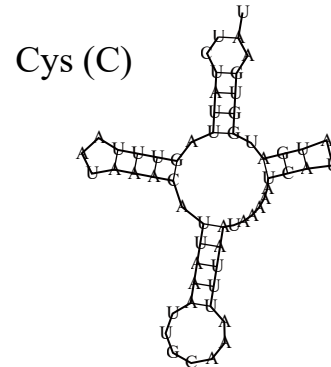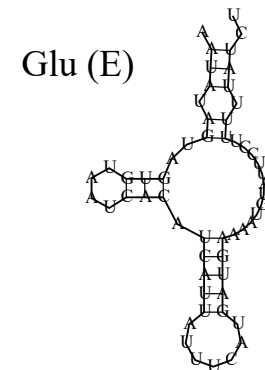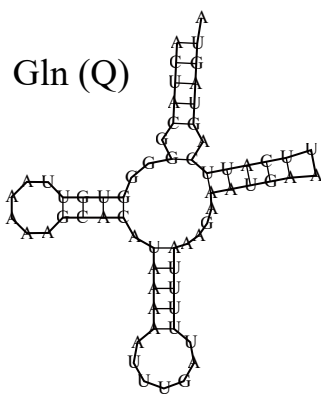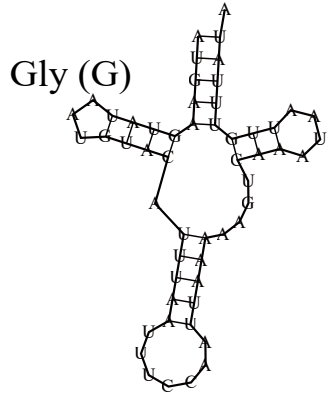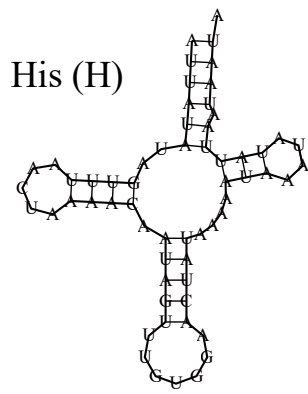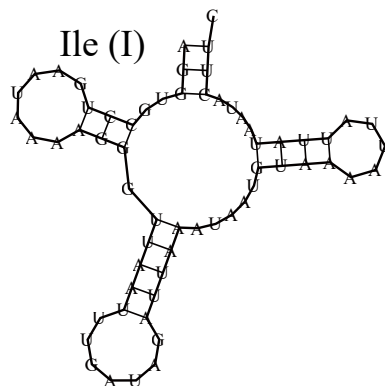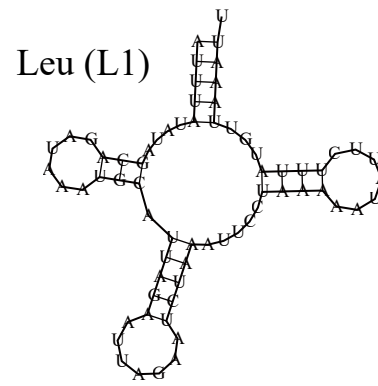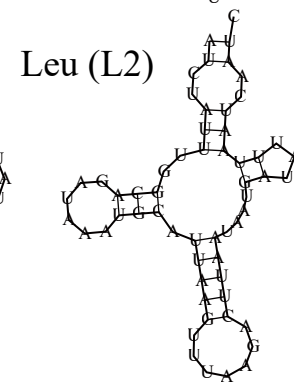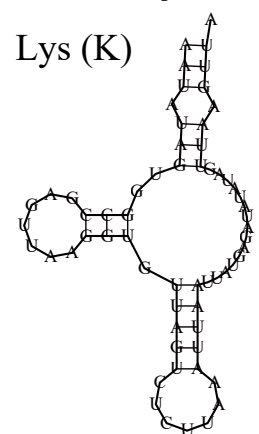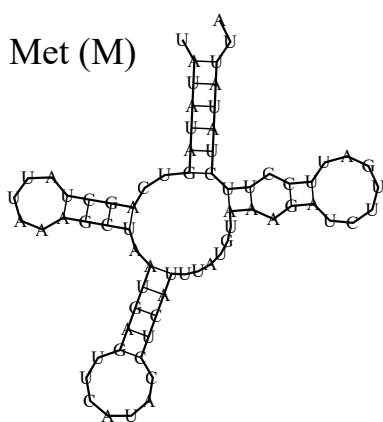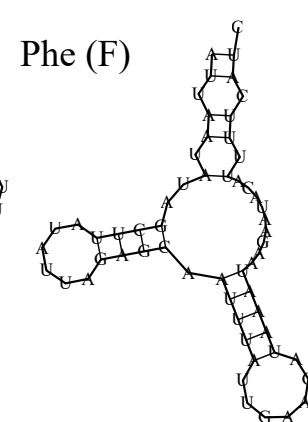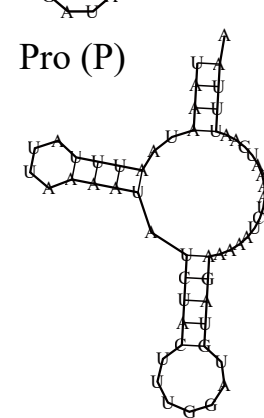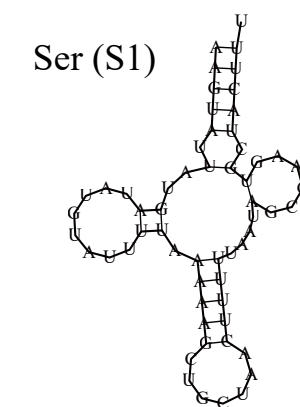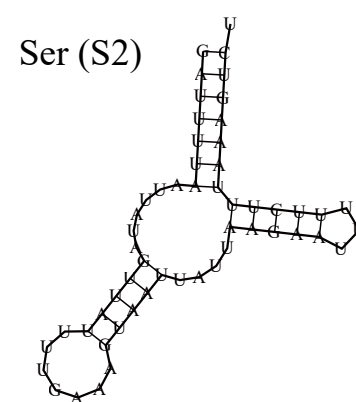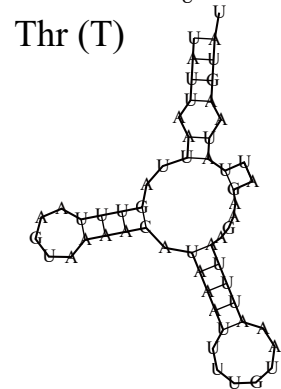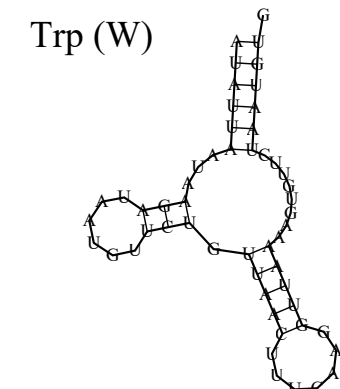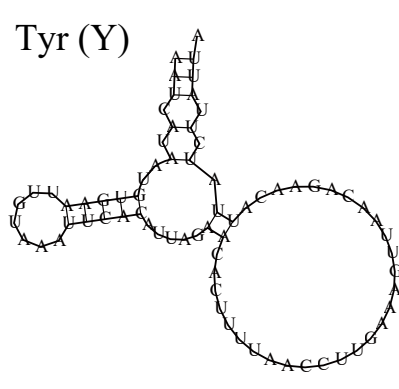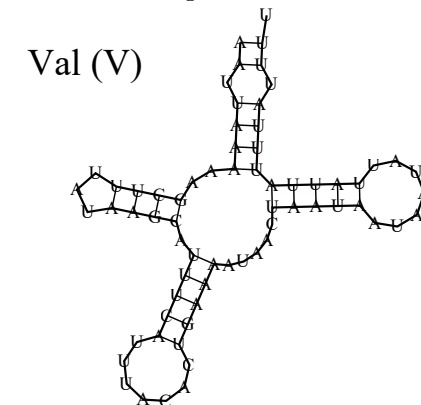

Supplement: Supplemental Information 7 — The tRNAs are labeled with the abbreviations of their corresponding amino acids. [file peerj-13-18314-s007.pdf]

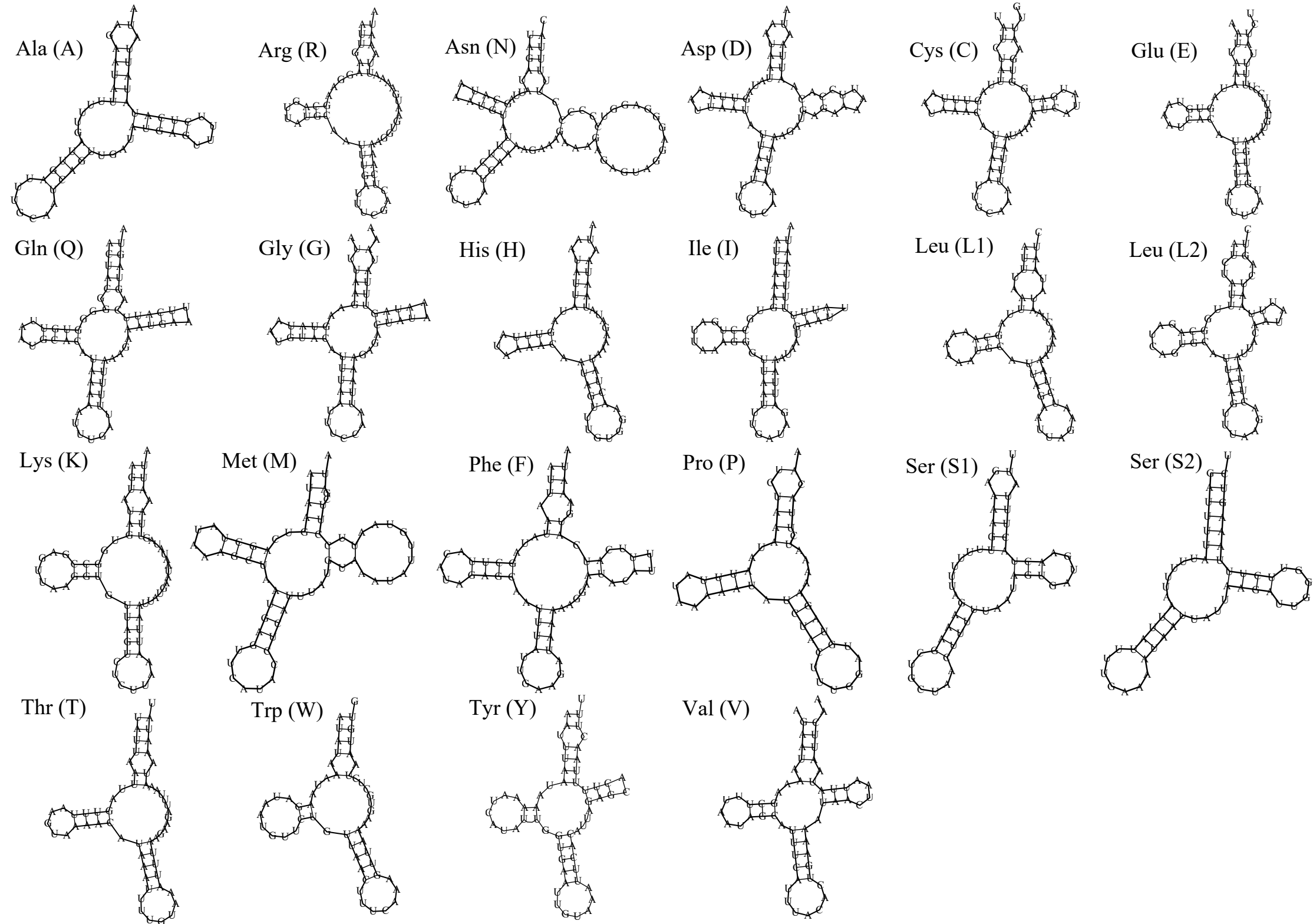

Supplement: Supplemental Information 8 — The tRNAs are labeled with the abbreviations of their corresponding amino acids. [file peerj-13-18314-s008.pdf]

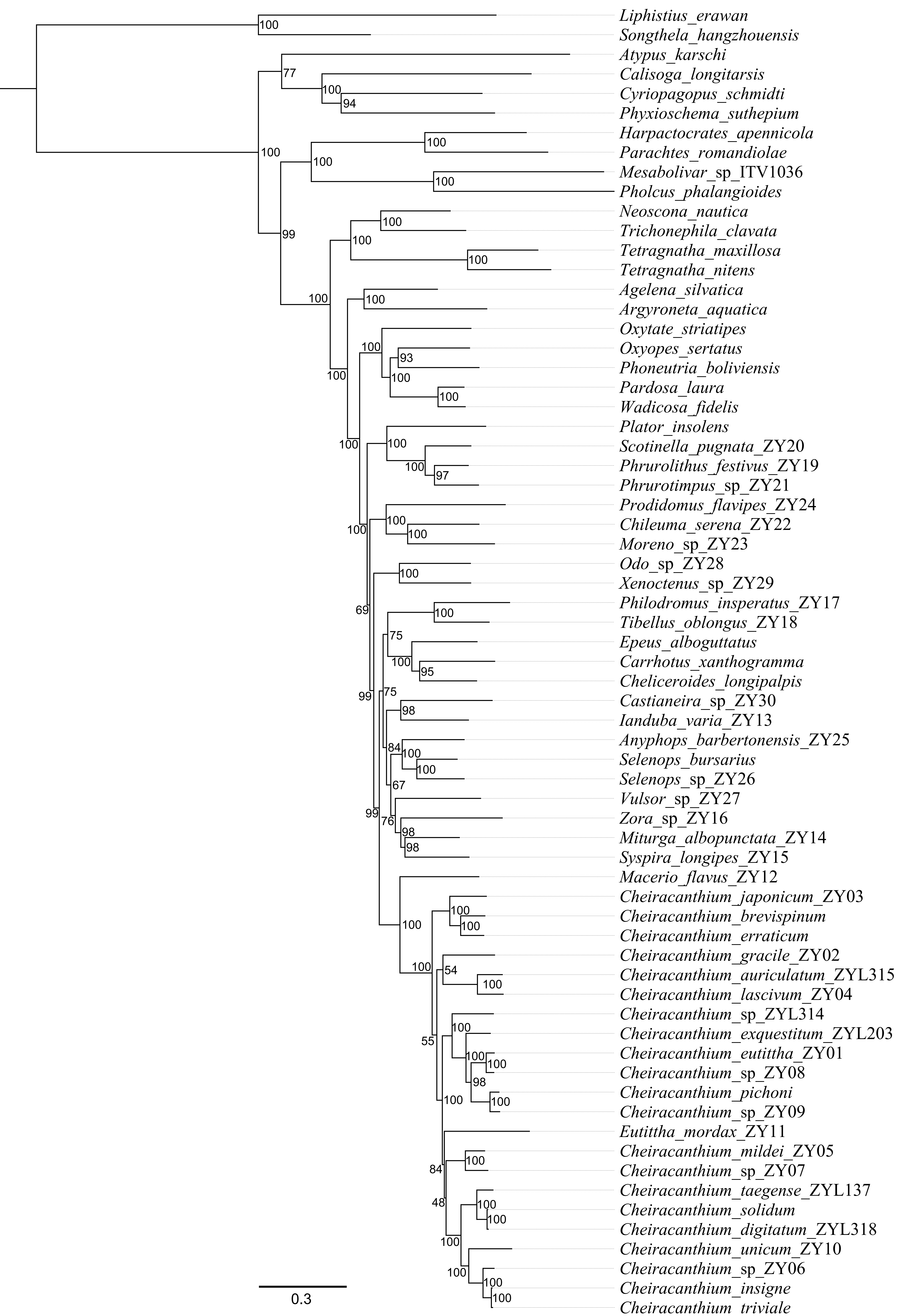

Supplement: Supplemental Information 9 — The relationships were constructed based on nucleotide sequences of 13 protein-coding genes. The numbers at the nodes are bootstrap values. [file peerj-13-18314-s009.pdf]

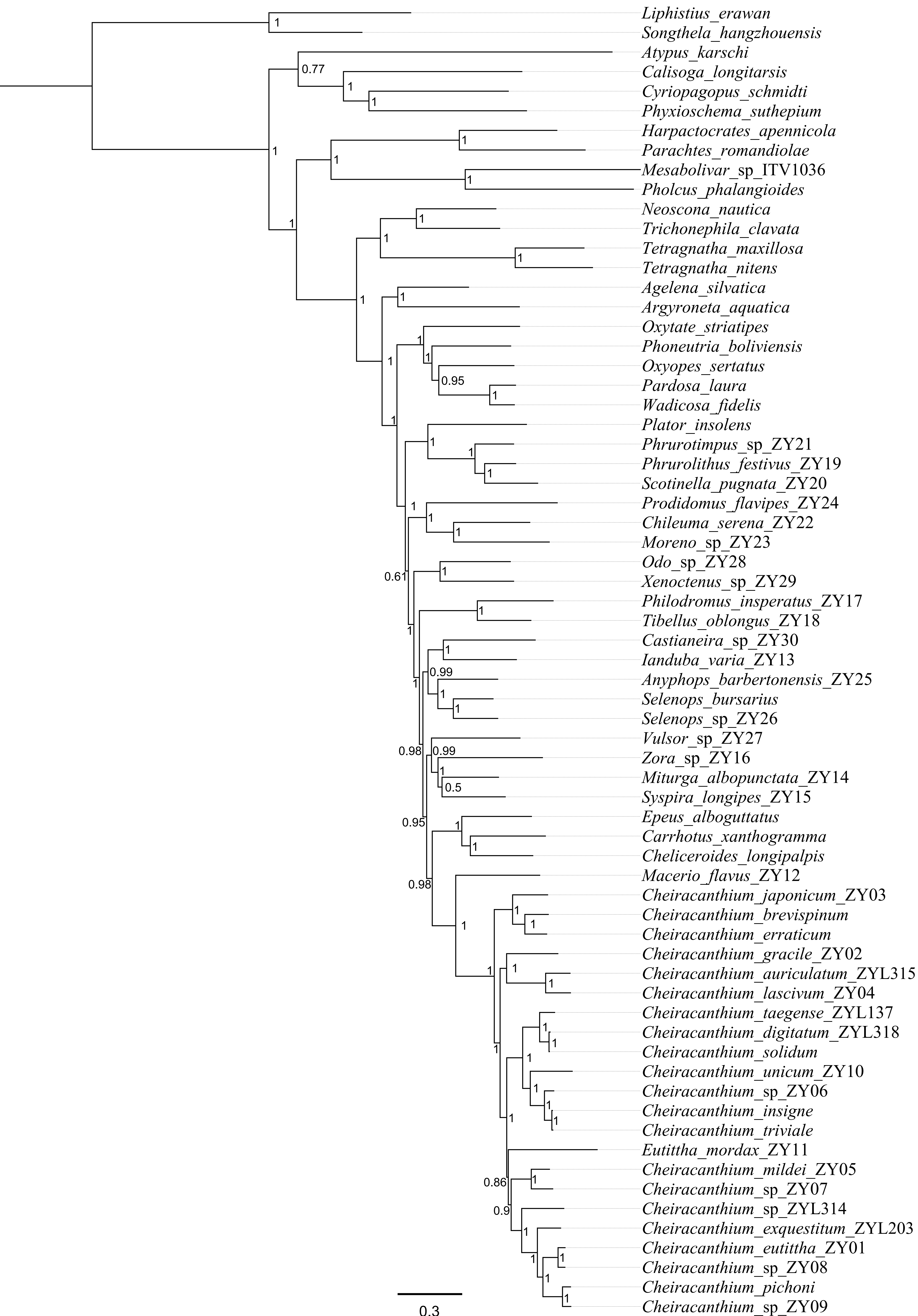

Supplement: Supplemental Information 10 — The relationships were constructed based on nucleotide sequences of 13 protein-coding genes. The numbers at the nodes are Bayesian posterior probabilities. [file peerj-13-18314-s010.pdf]
